# Virulence and Pathogen Multiplication: A Serial Passage Experiment in the Hypervirulent Bacterial Insect-Pathogen *Xenorhabdus nematophila*

**DOI:** 10.1371/journal.pone.0015872

**Published:** 2011-01-31

**Authors:** Élodie Chapuis, Sylvie Pagès, Vanya Emelianoff, Alain Givaudan, Jean-Baptiste Ferdy

**Affiliations:** 1 The Center for Biology and Management of Populations, Montpellier, France; 2 Laboratoire EMIP, UMR INRA UM2 1133, Université Montpellier 2, Montpellier, France; 3 Laboratoire Évolution et Diversité Biologique UMR CNRS UPS 5174, Université Toulouse 3, Toulouse, France; University of South Florida College of Medicine, United States of America

## Abstract

The trade-off hypothesis proposes that the evolution of pathogens' virulence is shaped by a link between virulence and contagiousness. This link is often assumed to come from the fact that pathogens are contagious only if they can reach high parasitic load in the infected host. In this paper we present an experimental test of the hypothesis that selection on fast replication can affect virulence. In a serial passage experiment, we selected 80 lines of the bacterial insect-pathogen *Xenorhabdus nematophila* to multiply fast in an artificial culture medium. This selection resulted in shortened lag phase in our selected bacteria. We then injected these bacteria into insects and observed an increase in virulence. This could be taken as a sign that virulence in *Xenorhabdus* is linked to fast multiplication. But we found, among the selected lineages, either no link or a positive correlation between lag duration and virulence: the most virulent bacteria were the last to start multiplying. We then surveyed phenotypes that are under the control of the flhDC super regulon, which has been shown to be involved in *Xenorhabdus* virulence. We found that, in one treatment, the flhDC regulon has evolved rapidly, but that the changes we observed were not connected to virulence. All together, these results indicate that virulence is, in *Xenorhabdus* as in many other pathogens, a multifactorial trait. Being able to grow fast is one way to be virulent. But other ways exist which renders the evolution of virulence hard to predict.

## Introduction

Historically, our understanding of how virulence evolves in pathogens has been marked by two trends. First, scientists have recognized that virulence could impede pathogen transmission because it reduces the capacities of an infected individual to contact susceptible conspecifics. This has led to the so called “avirulence theory” (see [1] for a historical perspective on this point). This theory has been challenged in the eighties by the seminal papers of Anderson and May [2], [3] and of Ewald [4]. These authors have proposed that the virulence of a pathogen, quantified as the increase in hosts' death rate caused by the infection, was positively correlated to its capacity to infect a susceptible host. They further assumed that the link between these two life-history traits was following the law of diminishing return: the benefit of an increased virulence, in terms of gained power to contaminate, decreases with virulence. Thus, pathogens can be poorly transmitted either because they kill their host before they have a chance to contaminate other individuals, as stated by the avirulence theory, or because they are not contagious enough. Under these hypotheses, they showed that pathogens should evolve toward low, but non null, virulence, a prediction that matches most empirical observations (see again [1] for a more detailed historical perspective). This proposal has since then be called the “trade-off hypothesis”.

Since the first mention of the trade-off hypothesis, many theoretical studies have surveyed how various changes in the initial model of Anderson and May [3] could affect the evolution of pathogen virulence. All these models rely on some sort of trade-off between virulence and transmission. Meanwhile, little progress was made in actually demonstrating the trade-off hypothesis in a natural system. Today, the empirical evidences that support the model of [3] exist, the historical demonstration being provided by the case of myxoma virus in European rabbit [5], but are still scarce (see [1] for a review). This situation probably explains the recent controversy about the trade-off hypothesis [6].

One of the most classically invoked justification of the trade-off hypothesis is the idea that both virulence and transmission increase with parasitic load. If this assumption holds, any trait that affects how fast a parasite multiplies in its host should have an impact on virulence. Some of these traits should be specific to the within host environment. Traits that allow pathogens to escape from their host's immune system would be among those. Other traits could relate to a general capacity to grow fast, irrespective to the environmental condition the pathogen lives in.

In order to better understand how selection on bacterial traits that affect growth kinetics impacts virulence, we conducted a serial passage experiment in *Xenorhabdus nematophila*. This bacterium is a hyper-virulent insect pathogen that is, in nature, transmitted by a nematode vector, *Steinernema carpocapsae* [7]. Our selection procedure was designed so as to impact bacteria multiplication in an artificial culture medium. A classic expectation in such experiments is that pathogens' adaptions to their hosts are disrupted by selection to adapt to the artificial environment, so that their virulence is attenuated [8]. We found that, contrary to expectation, the bacterial lineages we selected are more virulent than their common ancestral lineage. The genetic basis of *Xenorhabdus* virulence is not yet fully understood. Gene inactivation studies have identified regulatory proteins that alter virulence in insects and directly or indirectly affect expression of the flagellar regulon [9]–[12] The master regulator for flagellar synthesis and motility, FlhDC, has been shown to be required for full virulence in insects and for haemolysin and lipase production [9]. We found that the expression of some of these traits were changed in our selected lineages but that these changes did not correlate to changes in virulence. Overall, our work demonstrate that the determinism of *Xenorhabdus* virulence is multi-factorial so that virulence can be accidentally increased by selection in artificial conditions.

## Materials and Methods

### Biological material

In nature *X. nematophila* is an insect pathogen that develops in its host haemolymph. But it can also easily be cultivated in artificial culture medium. For example generation time in agitated liquid Luria Bertani medium (LB) at 28°C is about 80 mn for strain Be06. *Xenorhabdus* can also be conveniently cultivated onto NBTA plates (nutrient agar plates supplemented with 25 mg of bromothymol blue per liter and 40 mg of triphenyltetrazolium chloride per liter, see [13]). On this particular solid culture medium, *Xenorhabdus* colonies have a typical violet-blue color, which allows to distinguish them from potential contaminants.

In this work, we used *Xenorhabdus nematophila* (Enterobacteriaceae) strain Be06 as the ancestral lineage of our selection experiment. This particular strain has not been studied as extensively than the strains AN6 or F1 of the same species. It has been isolated in 2001 in a *Steinernema carpocapsae* nematode from Belgium and stored at −80C with 15% glycerol since then [14]. We thus have little knowledge on the particular behavior of this strain, compared to other more studied strains, but we have the guaranty that it has not experienced many generations of selection in laboratory conditions.

### Step one: experimental selection on bacteria

In our experiment, two selection treatments were imposed on bacteria. In one treatment, bacteria were transferred on fresh medium every 24 h with a dilution factor of 1∶10000. This is identical to the selection treatment used by [15]. In their work on *Escherichia coli*, these authors have shown that, under these conditions, cultures are not saturated within the 24 h of growth. Bacteria thus experience only the exponential phase of their growth. They found that this particular environment selects bacteria with larger cell size, which probably enables them to start growing faster when they are transferred in fresh culture medium [16]. In our experiment, we thus expect that bacteria experiencing this treatment will be selected for shorter lag phase and/or higher growth rate.

In a second treatment, bacteria were also transferred on fresh medium every 24 h but with a dilution factor of 1∶10. Under this condition, density was kept high during the whole 24 h culture periods, so that bacteria should probably not experience any exponential growth phase. We expect that bacteria experiencing this treatment should be selected on their capacity to multiply at high density.

In the following, for the sake of simplicity, we will call the first treatment Low Density Inoculum (LDI) and the second High Density Inoculum (HDI). Each selection treatment was applied to 40 independent selection lines that all originated from the same ancestral clone of strain Be06. The 80 lines were placed in four 96 cells tissue culture plates (FALCON), with the 20 lines of a plate experiencing the same selection treatment. The remaining empty cells of each plate were all filled with LB, which allowed us to check that cross-contamination between cells of the same plate did not occur during the course of the experiment.

During the selection experiment, culture plates were placed in a shaking incubator at 28°C for 24 h. After each 24 h period, cultures were transferred on plates containing fresh culture medium. The volume of all cultures was fixed at 200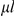. In LDI treatment, transfers were performed using a multi-channel pipet, by first transferring 0.2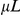 of each bacterial culture into 198 of fresh medium, and transferring again 0.2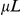 of this dilution into 198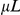 of fresh medium. In HDI treatment, transfers were performed by pipeting 20 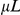 of bacterial culture into 180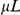 of fresh medium. We performed 40 cycles of culture and transfer for all lines. The whole selection experiment represents approximately 200 bacterial generations in LDI conditions. Samples from the last transfer were then stored at −80C with 15% of glycerol in individual cryotubes.

### Step two: measuring bacteria life history traits

After the last transfer, we measured life-history traits on each of the 80 selected lineages. We give below a list of those measurements and of the statistical methods we used to analyze them. All statistical analyses have been performed using the R statistical software [17].

#### Measuring bacterial growth

We studied bacterial growth by placing the ancestral and selected lines under identical culture conditions and by measuring how absorbance varies over time. To that purpose, a pre-culture was first initiated from bacteria kept in cryotubes. This bacteria were placed in liquid LB at 28°C for 24 hours. The pre-culture was then transferred into a micro-plate with a 1∶1000 dilution factor and absorbance was measured over a 20 hours culture period using a spectrophotometer (TECAN). During this 20 hours period, cultures were agitated and temperature was fixed to 28°C. Absorbance measures were taken each 15 minutes. After the last absorbance measurement was taken, we estimated the final number of bacteria by plating a 1∶100000 dilution of the culture on NBTA plates and by counting Colony Forming Units (CFU) on the plates after 48 h-incubation at 28°C. This experiment was replicated three times.

When placed in fresh culture medium, bacteria do not start multiplying immediately [18]. The lag before bacteria cells start dividing is of particular interest to us, as it as proved to be under strong selective pressure in former work [15], [16], [19]. We estimated the duration of this lag by measuring the time at which a culture over-reaches the absorbance of control wells that contain only sterile culture medium. More precisely, for each plate, the threshold absorbance was fixed as 1% over the maximum absorbance of the 200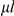 cells that contain LB. For each selection line, we thus obtained three independent estimates of this lag time. We analyzed this data using the Cox proportionnal hazard model, which is a non-parametric survival analysis tool. This was performed using the coxme function [20] in R. In this model we incorporated the first measured absorbance as a covariate, so that differences in the initial number of bacterial cells do not influence our analysis. We ignored the interaction between these two explanatory variables, as it proved to be statistically non significant. The possible differences between the three replicate experiments were modeled as a random block effect.

We used a similar statistical tool to estimate a median lag time for each selected line. This estimate is the time that elapsed before 50% of the cultures of a given selected line reach the threshold absorbance. This estimation was performed by adjusting a parametric survival model to our data, using the survreg function [21] in R. The parametric model we used has the same fixed and random factors than the non-parametric model we described above. A Weibull distribution of lag time was assumed, as this choice proved to maximize the model likelihood.

We analyzed other aspects of bacterial growth in LB by inspecting how variation in absorbance (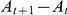) relate to current absorbance (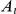). If growth was exponential, this link would be linear. If growth was logistic, the link would be quadratic. In our case, we found that none of these simple models adequately describe our data. We therefore used a Generalized Additive Model to identify differences in growth curve between bacteria of our two selection treatments and the ancestral lineage. This method allowed us to adjust smothing functions to the kinetics obtained for each selection treatment. This was performed using the mgvc library [22] in R. We chose a spline smoothing function and incorporated a temporal autocorrelation random term in our model. In order to test for a difference in growth curve between selection treatments, we compared a model where the relation between 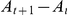 and 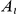 is adjusted for each treatment to a model where the same relation is adjusted for all treatments combined. The two models were then compared using a likelihood ratio test with four degrees of freedom, corresponding to the four additional parameters that are estimated in the first model.

#### Bacteria virulence towards *Galleria mellonella*

*Xenorhabdus* are known to be virulent against a wide variety of insect species. In laboratory conditions Lepidoptera are often used as convenient experimental hosts. Among those *Galleria mellonella* (Lepidoptera, Pyralidae) is known to be weakly resistant to infection. In our work, we used *G. mellonella* that were reared in the University of Montpellier (France). Insects were fed on pollen and wax, at 28°C in the dark and in aerated glass jars. The bacterial pathogenicity was measured by injecting a bacterial suspension representing approximately 2000 bacteria cells into *Galleria* last instars [23] and measuring insect survival over 46 hours. Bacterial cells were obtained from stationary phase cultures, which absorbance was used to adjust dilution. Appropriate volumes of the culture were diluted in PBS (phosphate-buffered saline) and dose was confirmed *a posteriori* by plating this dilution. In two independent series, we injected a total of 8 insect larvae for each bacterial strain. 81 different strains were studied this way: the 80 selected lines, and the ancestral line for which five independent replicate measurements were performed. We used as a negative control a total of 84 larvae that were injected with sterile PBS.

After injection, the number of injected bacteria was controlled by plating appropriate volumes of the injected dilution on NBTA plates and counting CFU after 48 h at 28°C. The weight of each *Galleria* larvae, which can potentially affect survival, was also measured before injection.

After injection, insect larvae were placed in an individual well of a 24 cells culture plate (FALCON) at 24C. A first measure of survival was performed 18 hours after injection, a time at which healthy insect larvae should survive. From 25 to 30 hours after injection, insect survival was then checked every 30 minutes, which yields 11 checking points. A final measure was then done 46 hours after injection, a time at which all insects injected with bacteria should be dead.

As for lag time, we analyzed the time at which insects died using a non-parametric survival analysis tool. We included the weight of the insect and the number of CFU of the infected culture as covariates, in order to reduce their possible effects on our test. We also considered the possible differences between the two insect lots we used as a random block factor.

#### Bacteria phenotypic traits

The super regulon flhDC is involved in bacterial virulence towards insects [9]. We thus measured motility, haemolysis, and lipolitic activity, that are all Flhdc dependent properties [9]. We also measured *Xenorhabdus* antibiotic activity, which is not Flhdc dependent. This property is important in natural conditions but should not be submitted to strong selection in our experimental design. Contrasting changes in antibiotic activity to changes on the other properties we have measured will thus allow us to test the hypothesis that selection has played specifically on Flhdc dependent traits.

We measured phenotypes on a random subset of 10 LDI and 10 HDI selected lines, as described in [9]. In a first experiment we measured, for each of these 20 lineages, motility and antibiosis on approximately 100 clones issued from two overnight cultures (the total number of clones measured being 2241). Bacterial motility was measured as the diameter of a halo motile bacteria form on 0.35% agar solid culture medium (see [24], [25]). Antibiotic activity was quantified by measuring the diameter of inhibition halos, using *Micrococcus* as a test organism [9], [26]. For that purpose, *Xenorhabdus* was cultivated at 28°C for 48 hours on Petri dishes containing 15.5 g of nutrient agar and then killed by exposing them to chloroform for 30 min. A *Micrococcus* culture was diluted by 2∶100 in fresh culture medium and streaked onto the plates which were then placed at 37C. Inhibition halos were measured after 24 hours of incubation. In a second experiment, we measured motility, haemolytic activity and lipolytic activity on at least 44 clones issued from a single overnight culture for each lineage (the total number of clones analyzed in this experiment being 1074). Extracellular lipase was indicated by a halo of precipitated material surrounding the colony cultured on Tween 40 agar [9]. The lipolytic activity of each strain was measured as the proportion of clones with some lipolysis. Haemolytic activity was assayed by cultivating bacteria on Trypticase soy (bioMerieux) with 5% (vol/vol) defibrinated sheep blood (bioMerieux). Haemolysis was then determined by the presence of a clearing surrounding bacteria grown on standard sheep blood agar plates [27], [28]. The haemolytic activity of each strain was assessed as the proportion of clones with no, partial, weak or total haemolytic activity.

As bacteria motility is measured twice and as some of the bacterial traits are correlated, we combined all our measurements in a multivariate analysis to extract independent measures that each quantify one phenotypic particularity of our bacteria. For that purpose, we had first to transform motility and antibiosis, which are quantitative, into qualitative measures. We defined a first class corresponding to clones with no halo (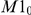 and 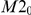 being the propotion of non-motile clones in the two replicate experiments while 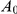 is the proportion of clone with no antibiosis halo). Other clones were assigned to in three distinct classes, corresponding respectively to clones with halo diameter below the first quartile (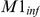, 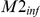, 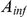), clones with halo diameter above the third quartile (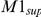, 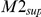 and 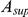) and clones with halo diameter comprised between the first and the third quartile (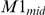, 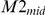 and 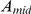). All these measurements thus make up 18 variables that each correspond to a proportion of clones with a specific phenotypic characteristic. We analyzed these 18 variables, using a scaled Principal Component Analysis. Each variable was assigned a weight corresponding to the inverse of the number of phenotypic classes for the corresponding measurement. Thus a 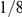 weight was given to all classes for motility and 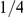 for antibiotic measurement and haemolytic activity. A 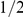 weight was assigned to the two variables describing the lipolytic activity. The analysis was performed using the ade4 library [29] in the statistical software R.

From this analysis, we also computed a measure of how each lineage has diverged from the ancestral lineage. This has been done by computing the distance between the point corresponding to the ancestral lineage and that of each selected lineage in the plane described by the first two axes of the PCA.

## Results

### Selected bacteria start growing faster than the ancestral lineage

**Figure 1:**
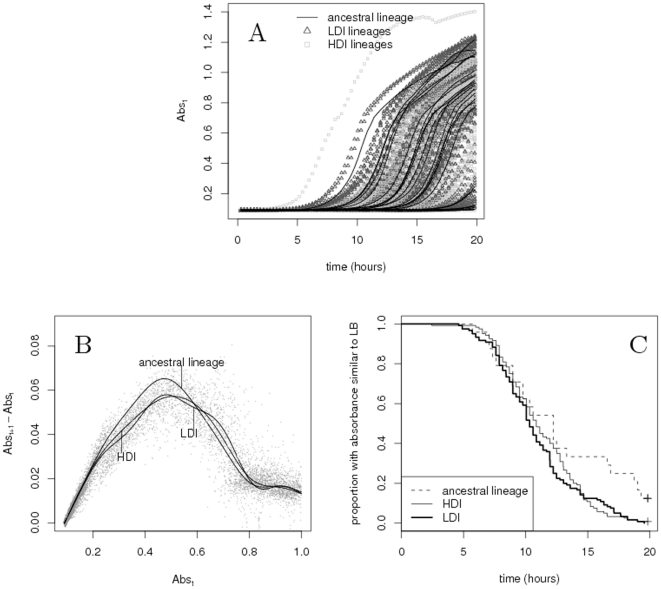
**A.** The absorbance (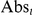) of each culture as a function of time. For each curve, we analyzed separately the time that elapses before the absorbance starts increasing and the variation in absorbance after this first increase has occurred. **B.** In spite of the great variance observed in A, the shape of the growth curve each culture follows is fairly stereotyped. This is represented here by the humped relationship that exists between the increase in absorbance (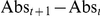) and current absorbance (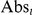). The curves in this figure represent the prediction of a GAM adjusted for each treatment on all replicate experiments (see text). The maximum increase in absorbance is significantly lower and occurs for higher absorbance in selected bacteria than in the ancestral lineages. **C.** Proportion of cultures which absorbance has not yet increased above that of wells containing only LB, as a function of time, for the ancestral lineage, the HDI treatment and the LDI treatment lines. This time varies a lot from one culture to another, but always positively correlates to the number of cells present in the culture after 20 hours. Overall, we found that absorbance starts increasing sooner in selected bacteria than in the ancestral lineage, although this difference was not found in one of the three replicate experiments.

We have monitored bacteria density over a 20 hours period in three replicate experiments, as illustrated in figure 1A. One of the most striking result from these measurements is that the time at which absorbance starts increasing varies tremendously between replicate cultures. Conversely, after absorbance has started increasing and with few exceptions, kinetics are rather stereotyped. We thus analyzed separately these two stages of bacteria kinetics.

Figure 1B depicts the kinetics after the first increase in absorbance has been detected. It represents the variation in absorbance as a function of current absorbance. The relationship between these two quantities is humped, with a maximum increase when absorbance is close to 0.5. We adjusted a General Additive Model (GAM) to these dynamics for each of the selection treatment. This revealed that selected bacteria differ from the ancestral lineage in several aspects of their growth curve (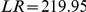, 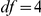, 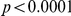). One of the clearest difference is that when absorbance exceeds 0.2, the increase is much lower in selected bacteria than in the ancestral lineage (see figure 1B). This can be tested by comparing between treatments the absorbance for which the increase is maximum. We found that the maximum increase is always reached for higher absorbance in selected bacteria compared to the ancestral lineage (Kruskal-Wallis test: 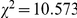, 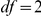, 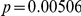, 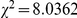, 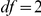, 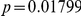 and 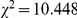, 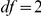, 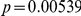 for the three replicate experiments respectively).

We then analyzed the time that elapses before the first increase in absorbance is detected. Figure 1C represents, as a function of time, the proportion of cells on a plate which absorbance has not yet increased above that of wells containing no bacteria. We found that this proportion drops faster in selected lineages than in the ancestral lineage, as demonstrated in table 1, with no significant difference between LDI and HDI lines. Still, fluctuations among replicate experiments are large (the estimated among experiment variance is 3.012 which is larger than the estimated variance among selection lineages). In fact, when results are analyzed separately for each experiment, significant differences among treatments are found only in the first two replicates.

**Table 1:** Analysis of the time that elapses before absorbance starts increasing.

	Estimate	Std. Error	z value	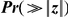
Initial absorbance	436.887	62.125	7.03	2.0e-12
HDI treatment	0.754	0.254	2.97	3.0e-03
LDI treatment	0.831	0.251	3.32	9.1e-04

This analysis was performed using a non-parametrical Cox model of survival. The total number of kinetics that are analyzed here is 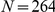. The variation between the three replicate experiments is modeled as resulting from a random factor. The possible influence of initial bacterial density is controlled by considering the initial absorbance as a covariate. This analysis shows that selected lines, LDI or HDI, reach the threshold absorbance faster than the ancestral lineage.

To confirm the fact that absorbance starts increasing faster in selected bacteria, we studied in more detail the kinetics of the ancestral lineage and of a subset of ten lines of each selection treatment. It is likely that the large variance we observed in the previous experiments is due in part to the fact that we do not control stricly the number of cells initially present in each bacterial population. We thus manipulated that number, for each of the 21 lineages, by diluting a single overnight culture by a factor 1∶100, 1∶1000, 1∶10000 or 1∶100000. After dilution, the bacterial populations were plated in order to estimate the actual initial number of CFU. Their absorbance was then followed over 20 hours of culture, as in the previous experiment. Two replicate experiments of this sort were conducted. The analysis first, summarized in table 2, shows that,as expected, the time that elapses before an increase in absorbance is detected decreases when the number of CFU initially present in the culture increases. Increases in absorbance were thus detected first in the least diluted culture. Most importantly, our analysis demonstrates that this effect is much stronger in selected bacteria than in the ancestral lineage. This confirms our previous conclusion that selected bacteria start growing earlier than the ancestral lineage.

**Table 2:** Analysis of the time that elapses before absorbance starts increasing for a subset of 20 selected lineages and with dilution factors ranging from 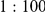 to 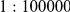 (the total number of kinetics that are analyzed here is 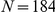, see text for further details).

	Estimate	Std. Error	z value	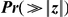
Initial CFU	2.288e-06	4.986e-07	4.59	4.5e-06
HDI treatment	5.958e-02	2.922e-01	0.20	8.4e-01
LDI treatment	-2.366e-01	2.949e-01	-0.80	4.2e-01
Initial CFU 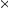 HDI treatment	2.124e-06	6.198e-07	3.43	6.1e-04
Initial CFU 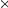 LDI treatment	2.743e-06	6.360e-07	4.31	1.6e-05

The analysis is conducted similarly to that presented in table 1. The initial number of CFU present in the culture, which decreases with the dilution factor, is used here as a co-factor. The kinetics of selected lineages is contrasted to that of the ancestral lineage and the variance among replicate experiments is modeled as a random factor (with estimated variance 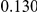). This analysis demonstrates that the time that elapses before we first detect an increase in absorbance decreases with the initial number of bacteria present in the culture. Most importantly, the two significant interaction terms indicate that this decrease is sharper in selected bacteria than in the ancestral lineage. We found no difference between the LDI and HDI lineages.

We found that selected bacteria start growing earlier than the ancestral lineage (see figure 1C). The biological significance of this result is relatively simple to demonstrate. We indeed found a significant correlation between the time at which we detected the first increase in absorbance and the number of CFU after 20 hours of culture in all three replicate experiments performed on the 80 selected lineages (Kendall correlation test, 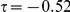, 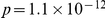, 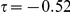, 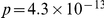 and 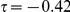, 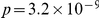) and in the dilution experiment (Kendall correlation test, 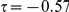, 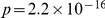). Our results therefore suggest that selected lineages are capable to reach high density faster than the ancestral lineage.

### Selected bacteria are more virulent than the ancestral lineage

**Figure 2:**
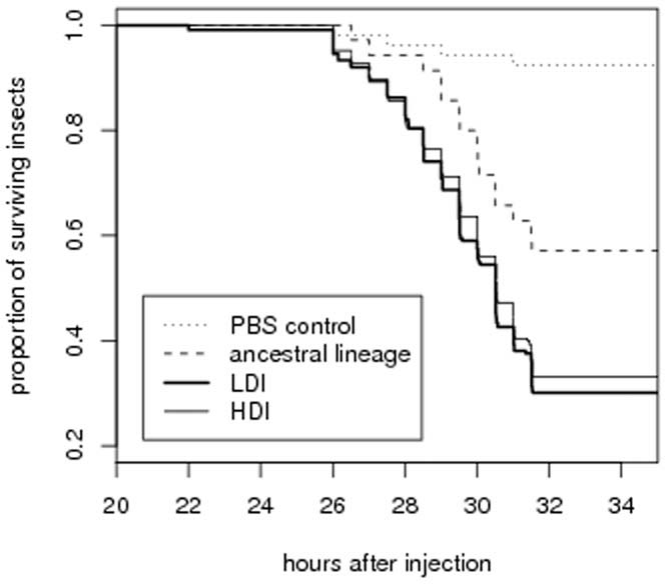
Proportion of surviving insects as a function of the time that elapsed since they were injected with bacteria. PBS injected insects serve here as a negative control, as they were not injected with bacteria. 46 hours after injection, more than 95% of all insects injected with bacteria were dead.

We measured bacteria virulence by injecting 2000 cells of each selection lines into *Galleria mellonella* last larval stage and by monitoring their survival. This moth species is highly susceptible to infection. A failed infection can thus be interpreted as resulting from the incapacity of the bacteria rather than from the resistance of the insect [30]. Figure 2 illustrates the fact selected lineages kill insects faster than the ancestral lineage. This is confirmed by the statistical analysis presented in table 3. Again, we found no statistically significant difference between LDI treatment and HDI treatment lines.

**Table 3:** Analysis of insect survival after they are injected with 2000 bacteria cells.

	Estimate	Std. Error	z value	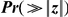
insect weight	−8.4461950	6.6008431	−1.28	0.2000
log(CFU)	−0.7317380	0.4378494	−1.67	0.0950
HDI treatment	0.4844258	0.2014537	2.40	0.0160
LDI treatment	0.549260	0.1988029	2.76	0.0057
insect weight 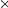 log(CFU)	4.1799256	3.1064825	1.35	0.1800

This analysis was performed using a non-parametrical Cox proportional hazard model of survival. In this analysis, we controlled for insect weight and for the number of injected CFU which were found to have no significant effect on survival. A total of 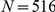 trials are analyzed here, with the differences between the lots of insects used in this experiment modeled as a random block factor (with an estimated variance of 0.008). The analysis demonstrates that insects injected with selected bacteria die earlier than those injected with the ancestral lineage.

### HDI bacteria have reduced FlhDC dependent phenotypes

**Figure 3:**
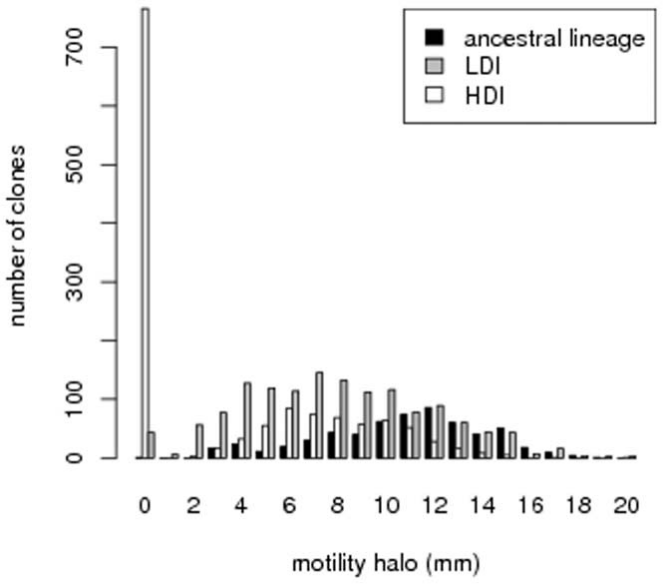
Distribution of motility halo diameter in the two selection treatments and the ancestral lineage.

In order to better understand what genetic changes might be responsible for the modifications we observed in bacteria virulence, we measured phenotypes that reveal the expression of genes under the control of FlhDC. These measures have been performed on the same 20 bacterial lineages that we used in the dilution experiment discussed above. We found that average motility is lower in HDI bacteria than in LDI bacteria in two replicate experiments (Wilcoxon test, 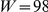, 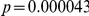 and 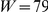, 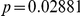 respectively, see figure 3). This difference is due to a massive increase in the proportion of non-motile clones in every HDI lineage, compared to the ancestral lineage: Fisher exact test performed on each selected lineage yields p values which are always lower than 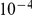 in the first experiment, while one lineage does not statistically differ from the ancestral lineage in the second experiment. When non-motile clones are excluded from the analysis, no difference can be found between LDI and HDI bacteria (Wilcoxon test, 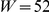, 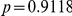 and 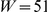, 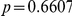 respectively). These results are illustrated in the left panel of figure 3, which presents the distribution of motility halo size for clones of all lineages and for the two experiments.

**Figure 4:**
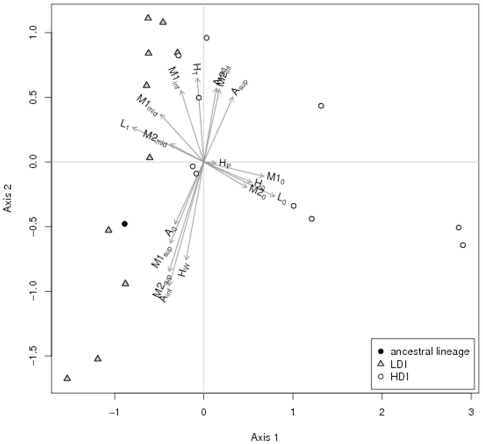
Principal component analysis of phenotypes for 20 of the 80 selected lines. See method for details on how this multivariate analysis was conducted. Most variation among HDI lineages occurs along the first axis, which strongly correlates with 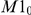 and 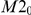, variable that correspond to proportions of non motile clones. It also correlates with 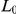, that measures the proportion of clones with no lipolytic activity, and 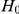, the proportion of clones with no haemolytic activity. Conversely, most variation among LDI lineages occurs mostly along the second axis. This axis correlates to the proportion of clones with total haemolysis and other variables indicating quantitative variation in motility and antibiotic production. This analysis therefore clearly demonstrates that LDI and HDI lineages have evolved differently.

We then analyzed all phenotypes using a multivariate approach (see figure 4). The two variables that correspond to the proportion of non-motile clones strongly correlate with the first axis of this PCA. As a result, the score on this axis is much higher in HDI than in LDI lineages (Kruskal-Wallis, 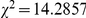, 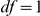, 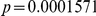). Variables indicating a loss in lipolitic activity (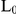) and in haemolytic activity (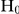) also correlate with this axis. This indicates that in HDI lineages clones that lack one of these three activities also lack the two others. It is therefore likely that FlhDC dependent functions are down regulated in most HDI lineages.

A massive reduction in motility is known to be a distinctive feature of variants of *Xenorhabdus* that can be obtained by a prolonged cultivation at high cell density [25]. Our observations could therefore indicate that the HDI selection treatment has enriched our bacterial populations in phase variants. In fact, in *Xenorhabdus* variants, the loss of motility is accompanied by a reduced haemolytic activity, a reduced production of antibiotics and by an increase in lipolytic activity [31], [32]. Here, we found a positive correlation between lack of motility and lack of lipolytic activity. We also found a negative, across treatment, correlation between the size of motility and antibiotic halos (Kendall's rank correlation, 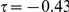, 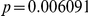). The same correlation, although marginally significant, is observed in LDI lineages (Kendall's rank correlation, 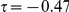 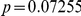). This correlation is not detected in HDI lineages, but we found that HDI lineages produce slightly more antibiotics than the ancestral lineage (mean antibiosis halo size is 8 mm and 9.79 mm for the ancestral lineage and the HDI lineages respectively, Kruskal-Wallis rank sum test 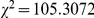, 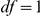, 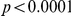). Overall, thus, we found no indication that a reduction in motility is accompanied, in our experiment, by a reduced antibiotic production. These observations indicate that the differences between the HDI bacteria and other lineages cannot be explained by an enrichment in phase two variants.

Compared to the first axis which quantifies the lack of three activities, the second axis of the PCA describes continuous variation in all phenotypes. Most variation among LDI lineages occurs along this axis. But some LDI lineages have scores that are lower than that of the ancestral lineage, while others have a higher score. LDI lineages have not evolved in fixed direction along this axis.

### The link between Flhdc dependent genes, lag period and virulence

Selected bacteria both start growing and kill insects earlier than the ancestral lineage. This result could be taken as a sign that virulence is linked by some cellular mechanism to the capacity of bacteria to start growing precociously. We tested this hypothesis on the same 20 lineages that we studied above, using the same type of analysis than that presented in table 3. In this analysis, we considered as an explanatory variable the time at which 50% of the bacterial populations of each lineage have overreached the absorbance of empty wells, which we shall for the sake of simplicity call the median lag time. Median lag time can be estimated with some accuracy on these 20 lineages, by using the 8 kinetics that have been measured for each lineage in the dilution experiment described earlier.

We also tried, in the same analysis, to relate FlhDC dependent phenotypes to the virulence of selected bacteria. We first chose to include the proportion of non-motile clones in the analysis, as we shown previously that this traits has rapidly evolved in some of the selected lineages (see figure 3). We also included the proportion of clones that display the total haemolysis phenotype, which relates to the production of the *xaxAB* protein. The production of another haemolytic protein *XhlBA* has indeed been shown by some authors to contribute to bacteria virulence [31]. Other traits have not been included both because of insufficient statistical power and because we showed in our multivariate analysis that they were strongly correlated to motility or haemolysis (see figure 4).

A correlation between a particular phenotype we have measured and virulence could indicate, first, that the genes that control the phenotype also impact bacteria virulence. But it could also be that, because of random sampling events, some bacterial populations have diverged more than others from the ancestral lineage. Those lineages should then both have divergent phenotypes and divergent virulence. In order to separate out these two potential source of variation, we considered the phenotypic distance between each lineage and the ancestral lineage as an explanatory factor.

**Figure 5:**
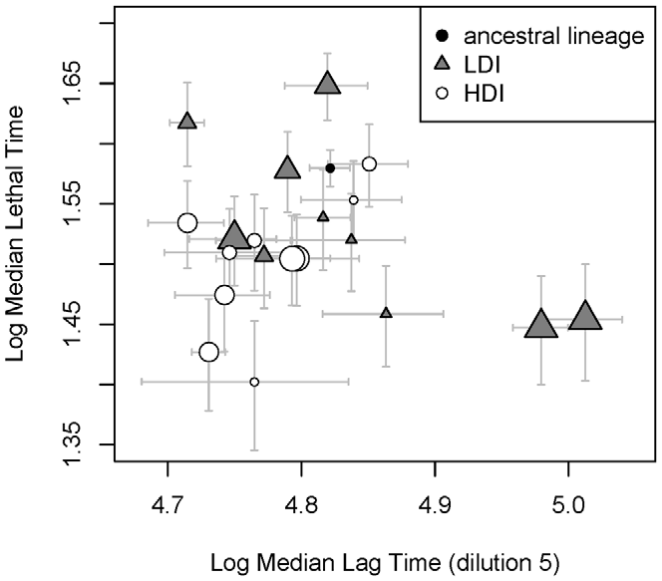
Median lethal time (i.e. the time at which 50% of the insects injected with a particular lineage are dead) as a function of median lag time (i.e. the time at which 50% of the populations of a particular lineage have overreached the absorbance of an empty well). The estimation of these times are performed using a parametric survival regression method (see method). Horizontal and vertical bars correspond to the standard error of these estimates. In LDI lineages, the two quantities are negatively correlated, indicating that the fastest growing LDI lineages are the least virulent. No relation could be found between medium lag time and medium lethal time in HDI lineages. The size of symbols indicates the proportion of clones that display the total haemolysis phenotype. When analyzing both ancestral and selected lineages, we found a marginally significant link between this variable and virulence.

When analyzing selected and ancestral lineages, we found no significant effect of median lag. In HDI bacteria, we found no correlation between any of our explanatory variables, including median lag time, and virulence. In LDI bacteria we found a positive link between median lag time and virulence. This link is also detected with a more basic correlation approach (Kendall's rank correlation, 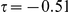 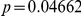). Contrary to our expectations, thus, the most virulent of the LDI bacteria are those which start growing the latest (see figure 5). We then analyzed the 40 LDI lineages, using the kinetics presented in figure 1 to estimate median lag time. As only three kinetics have been measured for each lineage in this experiment, median lag time estimates obtained this way are less accurate. We found a less clear negative relationship between the time necessary to kill an insect and this estimate of lag in LDI bacteria (Kendall rank correlation, 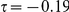, 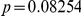) and no correlation at all in HDI bacteria (Kendall rank correlation, 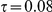, 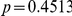).

Other variables that are tested in table 4 had no effect on virulence, except the proportion of clones with total haemolysis which we found to be marginally correlated to virulence when analyzing all lineages. This weak effect is not detected when using a simple rank correlation method (Kendall's rank correlation, 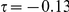 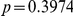). Overall, FlhDC dependent phenotypic traits seem to have little influence on bacteria virulence towards insects.

**Table 4:** Analysis of insect survival after they are injected with 2000 bacteria cells.

	Estimate	Std. Error	z value	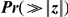
__________ **ancestral and selected lineages (N = 149)__________**				
Insect weight	−23.24	14.53	−1.6	0.11
Initial CFU	−0.7882	0.8706	−0.91	0.37
Median lag	1.545e−05	9.407e−06	1.64	0.1
Distance	−0.084	0.126	−0.67	0.5
Non-motile	0.6202	0.3709	1.67	0.094
Total haemolysis	0.7451	0.3769	1.98	0.048
Initial CFU 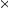 Insect weight	10.51	6.626	1.59	0.11
__________ **HDI lineages (N = 60)__________**				
Insect weight	16.33	43.26	0.38	0.71
Initial CFU	0.5323	3.049	0.17	0.86
Median lag	−0.2663	0.2292	−1.16	0.25
Distance	−0.1364	1.338	−0.1	0.92
Non-motile	0.1096	1.002	0.11	0.91
Total haemolysis	−6.493e-05	5.56e-05	−1.17	0.24
Initial CFU 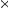 Insect weight	−7.991	19.16	−0.42	0.68
__________ **LDI lineages (N = 58)__________**				
Insect weight	−52.4	22.89	−2.29	0.022
Initial CFU	−1.788	1.223	−1.46	0.14
Median lag	4.057e-05	1.482e-05	2.74	0.0062
Distance	−0.1106	0.385	−0.29	0.77
Non-motile	−0.6982	2.365	−0.3	0.77
Total haemolysis	−0.3647	0.7414	−0.49	0.62
Initial CFU 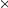 Insect weight	23.83	10.22	2.33	0.02

In this analysis, only the 20 lineages we used in the dilution experiment and for phenotypic measurements are present. In addition to the insect weight and the number of CFU present in the injected solution, we considered the median lag, the phenotypic distance to the ancestral lineage, the proportion of non-motile clones and the proportion of clones with the total haemolysis phenotype as explanatory variables. The analysis of all lineages show that the factor that explains the best the highest virulence of selected lineages is the increase in the proportion of total haemolysis, although this effect is marginal. The analysis of HDI lineages demonstrated no significant correlation between the virulence and the phenotypes we have measured. Conversely, in LDI lineages, we found a highly significant correlation between the virulence and the median lag time, i.e. the time that elapses before the absorbance of 50% of the bacterial populations of a lineage overreach that of empty wells. Contrary to our expectations, though, the LDI bacteria that kill insects the fastest are those which take the longest to start growing.

## Discussion

In this work we performed two types of serial passage experiments, with different dilution factors, in the bacterial insect pathogen *Xenorhabdus nematophila*. These passage experiments were performed *in vitro* and designed so as to modify bacteria multiplication. We found that, indeed, our selection treatments have changed bacteria kinetics in several different ways. In particular we found that the first increase in absorbance occurs earlier in selected bacteria than in the ancestral lineage. This observation is rather simple to interpret in biological terms, as we found that the bacterial populations that have the largest number of CFU after 20 hours of cultivation are those which absorbance started to increase first. This result therefore indicates that selected bacteria start multiplying earlier than the ancestral lineage, although the duration of the first phase of the kinetics varies a lot according to the initial number of cells, and maybe to some other environmental factors that may vary among inocula.

The duration of this first phase in fact combines the duration of the lag phase and the rate of bacteria multiplication during the very beginning of early exponential growth. We cannot say which of these two traits has varied during our experiment. Still, reduced lag time in the sort of serial passage experiment we performed has already been suggested for other bacteria [15], [16]. Some experimental data and theoretical arguments also suggest that mutations that reduce lag phase should be among the first to be fixed in serial passage experiments [19]. In fact, part of this reduction in lag phase might correspond to a simple adaptation to the artificial medium bacteria were cultivated in. This is supported by the fact that both LDI and HDI bacteria have a shortened lag phase, which indicates that some of selective pressures we exerted on bacteria during our serial passage experiment are the same in the two selection treatments.

We also found that selected lineages display a lower increase in absorbance when it exceeds 0.2. This result is very robust, as we detected this change in all the experiments we performed. Still, it is rather difficult to interpret. It is not clear, indeed, that this difference between the selected bacteria and the ancestral lineage is related to a modification in the rate of cellular division. Most of the *Xenorhabdus* strains are lysogenic [33], and a spontaneous phenomenon of bacteria lysis has already been reported in *Xenorhabdus* [34]. It is possible that the difference we observed between the selected bacteria and the ancestral lineage is due to a change in this sort of lysis phenomenon [35].

The most unexpected result of this study is that our selection procedure has also induced an increase in virulence. We indeed found that both LDI and HDI bacteria kill insects faster than the ancestral lineage. This was surprising to us, first because the virulence of *Xenorhabdus nematophila* is already very high. This was also unexpected because serial passage experiments that are performed on a particular host are generally found to increase virulence toward this specific host and attenuate virulence toward others [8]. By analogy, selecting bacteria in an artificial culture medium might disrupt some of the adaptations of the pathogen to its hosts, which should in turn lower its virulence.

In nature *Xenorhabdus nematophila* is adapted to grow in insect haemolymph, to compete with other bacteria within insect cadaver, and is transmitted by a nematode vector, *Steinernema carpocapsae* [7]. The ancestral strain we used in our experiment has been sampled in nature rather recently. Its behavior is thus probably shaped by all these constraints. In our selection experiment, bacteria did not compete with other pathogenic or scavenger microbes and did not have to reassociate with nematodes to ensure their transmission. Our results therefore indicates that when *Xenorhabdus* evolves without experiencing the constraints imposed by its transmission in nature, its virulence increases.

This can yield two different conclusions. First, even if *Xenorhabdus* is already very virulent, it must experience in its natural environment some selective pressures that prevent it from evolving towards even higher virulence. This would therefore be a sign that maximal virulence does not maximize transmission. This is in fact one of the predictions of the classic trade-off model for pathogen evolution [1]. In this model, indeed, it is assumed that there is a range of virulence for which the cost of an increased virulence, in terms of reduced opportunities of contact between the infected host and other individuals, outweighs the benefits, in terms of greater contagiousness of the pathogen. The mechanism that prevents *Xenorhabdus* from evolving toward even higher virulence must be different, as it is does not depend on its insect host for direct transmission. Still, it is possible that maximal virulence impacts negatively transmission, as *Xenorhabdus* has been shown to increase the mortality of its nematode vector [30], [36], [37].

A second conclusion comes from the fact that *Xenorhabdus* virulence can be increased by selection on traits that are expressed in artificial culture conditions. This would not be possible if the capacity of *Xenorhabdus* to kill an insect host was entirely determined by virulence factors that are specific to this host. A probable explanation of our results could thus be that bacteria kill insects by septicemia and that their virulence is determined in part by their capacity to start multiplying rapidly. This conclusion is also supported by the fact that *Xenorhabdus nematophila* is a generalist pathogen that is capable to kill a wild range of insect hosts [9], [11]. This conclusion can also be related to the trade-off model [1]. We saw before that one of the prediction of this model was that pathogens do not maximize their transmission by maximizing their virulence. The second prediction of this model is that the same pathogens will not either maximize their transmission by being totally avirulent. This is because this model assumes that the contagiousness of a pathogen increases with parasitic load, which comes at the expense of greater virulence. Here we found an indication that indeed greater multiplication capacity, which should determine parasitic load, results in greater virulence. Of course this interpretation should be considered with caution as we measured bacteria multiplication *in vitro*, and not in the insect host.

So far, we interpreted the fact our selected lineages have both a reduced lag phase and an increased virulence as a sign that any selective pressure that would increase the capacity of *Xenorhabdus* to reach high cell numbers in short times would also increase its virulence. This conclusion is in fact probably over-simplistic. First, phase variants are well described in *Xenorhabdus nematophila*. They are known to have a reduced lag phase (Givaudan, unpublished data) but they are not more virulent than wild type bacteria [9]. Second, in our experiment, we found no correlation between lag duration and virulence in HDI lineages and a strong positive correlation in LDI lineages: the most virulent of the LDI lineages are therefore those that start multiplying the latest. The reduction we observed in lag phase can therefore explain the difference between the ancestral and the selected lineages, but not the differences that exist among selected lineages. Clearly, virulence in *Xenorhabdus* is not uniquely determined by the duration of the lag phase or the capacity to multiply fast.

The difference we just mentioned suggests that the increase in virulence we have observed in both selection treatments has not been caused by the same genes in HDI and LDI lineages. The genetic determinism of virulence in *Xenorhabdus* is not fully understood. Nevertheless, the master regulator FlhDC is thought to play a role in *Xenorhabdus* virulence properties as flhDC mutants displayed delayed virulence after injection in insects [9], [11]. We therefore measured FlhDC dependent phenotypes in order to better understand what has determined the increase in virulence during our selection experiment.

Bacterial motility, which is determined by genes that control the expression of flagella components, is among these Flhdc dependent traits [9], [38]. We found that HDI bacteria have experienced a massive reduction in motility. This can be explained by the fact that, first, motility does not serve any purpose in the agitated artificial medium we cultivated bacteria in. Second, expressing flagella components clearly represents a high cost. It has recently been shown that *Xenorhabdus* express flagella components at high cellular density [39]. The cost of motility should therefore be much higher in HDI treatment, where bacteria are inoculated at high density, than in LDI condition, where density is initially low. This can explain why HDI but not LDI lineages have evolved towards reduced motility.

We also found that out of the 60 HDI clones that do not express lipolytic activity, 58 are non mobile. Similarly, most of the HDI clones which lack haemolytic activity also lack motility. Conversely, we found no link between the lack of antibiotic activity and the lack of motility in HDI lineages. Motility, lipolytic activity and haemolytic activity are under the direct control of the fhlDC super regulon while antibiotic production is not [40]. Our results therefore strongly suggest that our selection treatment has impacted the functioning of the flhDC regulon in HDI but not in LDI lineages. Statistical analysis, though, demonstrated no clear link between FlhDC dependent phenotype and virulence toward the insect. We only detected a weak positive correlation between the proportion of clones that display the total haemolysis phenotype and virulence. The FlhD-dependent haemolysin *xaxAB* has been found to strongly induce necrosis and apoptosis in immunocompetent insect cells *in vitro* [28], but the *xaxAB* mutant was shown to be as virulent as wildtype *X. nematophila* [38]. Thus, it is unlikely that *xaxAB* hemolysin is required for the full virulence of *X. nematophila*. Our results could be interpreted as contradicting these previous study, but we think that they are not robust enough to reach such a conclusion.

Measures of FlhDC dependent phenotypes provide a clear indication that LDI and HDI bacteria did not evolve identically. Of course, this does not necessarily mean that LDI and HDI lineages have experienced different selective pressures. The number of bacteria selected at each transfer was indeed a thousand times lower in LDI than in HDI treatment. Therefore, LDI lineages could have experience stronger bottlenecks than HDI lineages. Even if selection pressures were identical in the two treatments, faster genetic drift could have induced a different evolutionary trajectory in LDI lineages. This is in fact unlikely because, first, we found as much phenotypic variation in LDI than in HDI treatment. Second, assuming that an equilibrium population of *Xenorhabdus* contains approximately 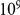 cells, 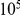 cells were transferred at each passage in the LDI treatment. This number is sufficiently high so that we can exclude the hypothesis that LDI lineages have evolved mostly by random genetic drift.

Our results lead to the conclusion that virulence, in *Xenorhabdus nematophila* as probably in many other pathogens, is a polygenic trait which link to fitness is complex (e.g. [41]). They also enlighten the great variance in how pathogens respond to selection. All these observations lead us to think that predicting how virulence evolves in response to even a simple selective pressure, of the sort we imposed in our experimental design, is among the most challenging tasks. We also demonstrated that a selective pressure in artificial conditions can accidentally increase the virulence of a pathogen. This is connected to the more general question of the emergence of hypervirulent pathogenic strains in anthropic environments [42]. To our opinion, combining the tools of microbiology and evolutionary biology as we did in this work is a promising way to better understand this issue.
